# Genomes of the T4-related bacteriophages as windows on microbial genome evolution

**DOI:** 10.1186/1743-422X-7-292

**Published:** 2010-10-28

**Authors:** Vasiliy M Petrov, Swarnamala Ratnayaka, James M Nolan, Eric S Miller, Jim D Karam

**Affiliations:** 1 Department of Biochemistry, Tulane University Health Sciences Center, 1430 Tulane Avenue, New Orleans, LA, USA; 2 School of Science and Technology, Georgia Gwinnett College, 1000 University Center Lane, Lawrenceville, GA 30043, USA; 3 Department of Microbiology, Campus Box 7615, North Carolina State University, Raleigh, NC 27695, USA

## Abstract

The T4-related bacteriophages are a group of bacterial viruses that share morphological similarities and genetic homologies with the well-studied *Escherichia coli *phage T4, but that diverge from T4 and each other by a number of genetically determined characteristics including the bacterial hosts they infect, the sizes of their linear double-stranded (ds) DNA genomes and the predicted compositions of their proteomes. The genomes of about 40 of these phages have been sequenced and annotated over the last several years and are compared here in the context of the factors that have determined their diversity and the diversity of other microbial genomes in evolution. The genomes of the T4 relatives analyzed so far range in size between ~160,000 and ~250,000 base pairs (bp) and are mosaics of one another, consisting of clusters of homology between them that are interspersed with segments that vary considerably in genetic composition between the different phage lineages. Based on the known biological and biochemical properties of phage T4 and the proteins encoded by the T4 genome, the T4 relatives reviewed here are predicted to share a genetic core, or "Core Genome" that determines the structural design of their dsDNA chromosomes, their distinctive morphology and the process of their assembly into infectious agents (phage morphogenesis). The Core Genome appears to be the most ancient genetic component of this phage group and constitutes a mere 12-15% of the total protein encoding potential of the typical T4-related phage genome. The high degree of genetic heterogeneity that exists outside of this shared core suggests that horizontal DNA transfer involving many genetic sources has played a major role in diversification of the T4-related phages and their spread to a wide spectrum of bacterial species domains in evolution. We discuss some of the factors and pathways that might have shaped the evolution of these phages and point out several parallels between their diversity and the diversity generally observed within all groups of interrelated dsDNA microbial genomes in nature.

## Background

Discovery of the three T-even phages (T2, T4 and T6) and their subsequent use as model systems to explore the nature of the gene and genetic mechanisms had a profound impact on the proliferation of interdisciplinary biological research. Indeed, work with these bacterial viruses during the period between 1920 and 1960 laid down several important foundations for the birth of Molecular Biology as a field of research that freely integrates the tools of almost every discipline of the life and physical sciences [1,2]. Phage T2, the first of the T-even phages to be isolated (see [3] for a historical perspective) occupied center stage in most of the early studies, although the underlying genetic closeness of this phage to T4 and T6 gave reason to treat all three phages as the same biological entity in discussions of what was being learned from each of them. The switch in attention from T2 to T4 came about largely as a response to two major studies in which T4 rather than T2 was chosen as the experimental system. These were the studies initiated by Seymour Benzer in the mid-1950s on the fine-structure of the phage *rIIA *and *rIIB *genes ( see [4] for an overview) and the collaborative studies by Richard Epstein and Robert Edgar [5] through which an extensive collection of T4 conditional lethal (temperature-sensitive and amber) mutants was generated [6] and then freely shared with the scientific community. Use of the Epstein-Edgar collection of T4 mutants, as well as comparative studies with T2 and T6 and other T4 relatives isolated from the wild, ultimately led to detailed descriptions of the structure, replication and expression of the T4 genome and the morphogenetic pathways that underlie phage assembly and the release of phage progeny from infected *Escherichia coli *hosts (see [2,7,8] for comprehensive reviews). As the best-studied member of this group of phages, T4 has become the reference or prototype for its relatives.

Over the last 50 years, hundreds of T4-related phages have been isolated from a variety of environmental locations and for a number of different bacterial genera or species [9,10]. The majority of these wild-type phages were isolated by plating raw sewage or mammalian fecal samples on the same *E. coli *strains that are commonly used in laboratories for growing T4 phage stocks or enumerating T4 plaques on bacterial lawns. The archived *E. coli *phages include both close and highly diverged relatives of the canonical T-even phages, as originally surmised from their serological properties and relative compatibilities with each other in pair-wise genetic crosses [11] and later confirmed through partial or complete sequencing of representative phage genomes [12-16]. In addition to the large number of archived T-even-related phages that grow in *E. coli*, there are several (<25) archived relatives of these phages that do not use *E. coli *as a host, but instead grow in other bacterial genera, including species of *Acinetobacter, Aeromonas, Klebsiella, Pseudomonas*, *Shigella, Vibrio *or photosynthesizing marine cyanobacteria ([9,10] and recent GenBank submissions, also see below). The sequencing of the genomes of a number of these phages has shown that they are all highly diverged from the T-even phages and that in general, there is a higher degree of genetic diversity among T4 relatives that are presumably genetically or reproductively separated from one another in nature because of their differences in the range of bacterial hosts they can infect [14-17]. The list of sequenced T4-related phage genomes has more than doubled during the last 3-4 years, further reinforcing the evidence for extensive genetic diversity within this group of phages. A major goal of the current review is to provide updated information about the sequence database for T4-related genomes and to summarize their commonalities and differences in the context of what is also being learned from the comparative genomics of other microbial organisms in nature. Ecologically, the lytic T4-related phages occupy the same environmental niches as their bacterial hosts and together with their hosts probably exercise major control over these environments.

### What is a T4-related or T4-like phage?

The International Committee for the Taxonomy of Viruses (ICTV) has assigned the T-even phages and their relatives to the "T4-like Viruses" genus, which is one of six genera of the Myoviridae Family http://www.ncbi.nlm.nih.gov/ICTVdb/index.htm. Broadly, the Myoviridae are tailed phages (order Caudovirales) with icosahedral head symmetry and contractile tail structures. Phages listed under the "T4-like Viruses" genus exhibit morphological features similar to those of the well-characterized structure of phage T4, as visualized by electron microscopy, and encode alleles of many of the T4 genes that determine the T4 morphotype [8]. The diversity of morphotypes among the bacterial viruses is staggering and to the untrained eye, subtle differences between different Myoviridae or different T4 relatives can be difficult to discern under the electron microscope [9,10]. In recent years there has been an increased reliance on information from phage genome sequencing to distinguish between different groups of Myoviridae and between different phages that can be assigned to the same group. The hallmark of the T4-like Viruses is their genetic diversity, which can blur their commonalities with each other, especially for taxonomists and other biologists who wish to understand how these and other groups of dsDNA phages evolve in their natural settings. As is the case for many other dsDNA phages, the genomes of T4 and its analyzed relatives are mosaics of one another, consisting of long and short stretches of homology that intersperse with stretches that lack homology between relatives [14-18]. Much of this mosaicism is thought to have resulted from DNA rearrangements, including genetic gains and losses ("indels"), replacements, translocations, inversions and other types of events similar to those that have shaped the evolution of all microbial genomes in nature. It appears that for the T4-like Viruses, DNA rearrangements have occurred rampantly around a core of conserved (but mutable) gene functions that all members of this group of Myoviridae encode. Sequence divergence or polymorphism within this functionally conserved core is often used to gain insights into the evolutionary history of these phages [16,19,20]. As the genome sequence database for T4 relatives has grown over the last several years, it has also become increasingly evident that the T4-like Viruses exist as different clusters that can be distinguished from one another by the higher levels of predicted genetic and biological commonalities between phages belonging to the same cluster as compared to phages in different clusters. Clusters of closely interrelated genomes have also been observed with other groups of dsDNA phages and microbial genomes in general, e.g., [21,22]. Many of the distinguishing features between clusters of T4-related phages are predicted to be the result of an evolutionary history of isolation within distinct hosts and extensive lateral gene transfer (LGT), i.e., the importation of genes or exchanges with a diversity of biological entities in nature. Genomic mosaicism, which appears to be a common feature of many groups of interrelated dsDNA phages [23,24], underscores the discontinuities that can be created by LGT between different lineages of the same group of interrelated phage genomes.

### The inventory of sequenced T4-related genomes

In Table 1, we have listed 41 T4-related phages for which substantive genome sequence information is currently available in public databases, particularly GenBank and http://phage.bioc.tulane.edu (or http://phage.ggc.edu). This listing highlights the bacterial genera and species for which such phages are known to exist [10] and includes recent entries in GenBank for three phages that grow in *Klebsiella, Pseudomonas *and *Shigella *species, respectively. The largest number of archived T4 relatives have originated from raw sewage or mammalian fecal matter and detected as plaque formers on lawns of laboratory strains of *E. coli *B and by using plating conditions that are particularly favorable for clear plaque formation by T4. *E. coli *K-12 strains have also been used in some cases (Table 1). The RB phages listed in Table 1 are part of the largest number of T4 relatives to have been collected around the same time from approximately the same environmental source. This collection consists of ~60 phages (not all T4-related) that were isolated by Rosina Berry (an undergraduate intern) from various sewage treatment plants in Long Island, New York during the summer of 1964 for Richard Russell's PhD project on speciation of the T-even phages [25]. The RB phages, which were isolated by using *E. coli *B as a host, include both close and distant relatives of the T-even phages and have received broad attention in comparative studies of the biochemistry and genetics of the T4 biological system [2,7,8]. The genomes of most of the distant relatives of T4 from this collection were sequenced and annotated several years ago [14-16]. More recently, draft or polished sequences have also become available for several close relatives of T4 from this collection as well as for phages T2 and T6 (see http://phage.ggc.edu for updates). The other phages listed in Table 1 are from smaller collections that originated through studies by various laboratories, as noted in the references cited in Table 1.

**Table 1:** An overview of sequenced T4-related phage genomes. ^(1)^

Bacteria	**Phages **^(2)^	Bacterial strain used in phage isolation
**Proteobacteria**		

*Enterobacteria*	T2, T4, T6	*E. coli *B (see [3] for references)

	RB3, RB14, RB15, RB16, RB18, RB26, RB32, RB43, RB49, RB51, RB70, RB69	*E. coli *B/5 [25]

	LZ2	*E . coli *B strain NapIV [62]

	JS8, JS10, JSE	*E. coli *K-12 strain K802 [69,74]

	CC31	*E. coli *B strain S/6/4 (Karam lab; New Orleans sewage, unpublished)

	phi1	*E. coli *K-12 F^+ ^(I. Andriashvili, 1971, unpublished); Tbilisi sewage; (M. Kutateladze pers. commun.)

*Acinetobacter*	133	*Ac. johnsonii *(see [14] for references)

	Acj9, Acj61	*Ac. johnsonii *(Karam lab; New Orleans sewage, unpublished)

	42 (=Ac42)	*Acinetobacter sp. *(H. Ackermann, D'Herelle Center, Canada; pers. commun.)

*Aeromonas*	44RR, 31, 25, 65	Various *Ae. salmonicida *strains (see [14] for references)

	Aeh1	*Ae. hydrophila *C-1 (see [14] for references)

	PX29	*Ae. salmonicida *strain 95-65 (Karam lab; New Orleans sewage, unpublished)

*Klebsiella*	KP15	*Klebsiella pneumoniae *(Z. Drulis-Kawa, pers. commun.; Warsaw, Poland sewage).

*Pseudomonads*	phiW-14	*Delftia acidovorance *(see GenBank Accession no. NC_013697)

*Shigella*	phiSboM-AG3	*Shigella boydii *(see GenBank Accession no. NC_013693)

*Vibriobacteria*	KVP40, nt-1	See [14] for references

**Cyanobacteria**		

*Synechococcus*	SPM2	*S. marinus *[27]

	S-RSM4	*S. marinus *[31]

	Syn9	*S. marinus*. Also grows in *Prochlorococcus *[75]

*Prochlorococcus*	P-SSM2, P-SSM4	P-SSM2, P-SSM4: [42]

^(1) ^The phages are listed under the major divisions (phyla) and genera of the bacterial hosts used for their isolation.

Each of the genomes we discuss in this review has a unique nucleotide sequence and a genetic composition that unambiguously distinguish it from the others. Yet, all of these genomes can be assigned to a single umbrella group based on shared homologies for a number of genes that we refer to here as the "Core Genome" of the T4-related phages, or T4-like Viruses. The genetic background for the Core Genome can vary considerably between T4 relatives and constitutes an important criterion for distinguishing between close and distant relatives among the ~40 phage genomes sequenced so far. The three T-even phages have traditionally been considered to be closely interrelated on the basis that they share ~85% genome-wide homology, similar genetic maps and certain biological properties in common with each other [8,26]. By using comparable criteria for phage genome organization and assortment of putative genes, i.e., predicted open-reading frames (ORFs) and tRNA encoding sequences, we could group the phages listed in Table 1 into 23 different *types *of T4 relatives, with the *T-even type *phages representing the largest group or cluster of closely interrelated phage genomes sequenced so far. These 23 *types *and their distinguishing features are listed in Table 2. The abundance of sequence data for the *T-even type *phages is largely the result of an effort by J. Nolan (in preparation) to analyze the genomes of RB phages that had been predicted by Russell [25] to be closely related to the T4 genome. We presume that in nature, each *type *of T4-related phage listed in Table 2 is representative of a naturally existing cluster or pool of closely interrelated phages that contains a record of evolutionary continuities between members of the pool. A pool of closely interrelated phages would be expected to exhibit low levels of sequence divergence between pool members, but might also show evidence of sporadic deletions, acquisitions, exchanges or other DNA rearrangements in the otherwise highly conserved genetic composition.

**Table 2:** T4-related phages with sequenced genomes

Phage or genome *type*	Phage	Genome size (bp)	**Database reference**^**(1)**^	ORFs (T4-like/Total)	tRNA genes	**Shared or unique properties of the genomes**^**(2)**^
***T-even***	*E. coli *phage T4	168,903	NC_000866	278	8	The *T-even type *genomes share 85-95% ORF homology with one another and >90% nucleotide sequence identity between most of their shared alleles. Also, these genomes encode glucosyl transferases and dCMP hydroxymethylases, but their DNA modification patterns vary (see text and Table 3). Some members of this cluster are known to be partially compatible with each other in genetic crosses (see text). Phage RB70 (Table 1) might be identical to phage RB51.
	*E. coli phage *T4T	168920	HM137666	280	8	
	*E. coli *phage T2	163,793	Tulane	232/269	9	
	*E. coli *phage T6	168,974	Tulane	228/270	7	
	*E. coli *phage RB3	~168,000	Tulane	~240/~270	10	
	*E. coli *phage RB14	165,429	NC_012638	235/274	10	
	*E. coli *phage RB15	~167,000	Tulane	~236/~269	7	
	*E. coli *phage RB18	166,677	Tulane	237/268	10	
	*E. coli *phage RB26	163,036	Tulane	232/~269	10	
	*E. coli *phage RB32	165,890	NC_008515	237/270	8	
	*E. coli *phage RB51	168,394	NC_012635	242/273	9	
	*E. coli *phage LZ2	>159,664	Tulane	~240/>260	10	

***RB69***	*E. coli *phage RB69	167,560	NC_004928	212/273	2	~20% of the ORFs in this genome are unique to RB69; this phage excludes T4 in RB69 × T4 crosses [25].

***RB49***	*E. coli *phage RB49	164,018	NC_005066	120/279	0	The 3 genomes of this *type *share 96-99% ORF homology with one another
	*E. coli *phage phi1	164,270	NC_009821	115/276	0	
	*E. coli *phage JSE	166,418	NC_012740	122/277	0	

***JS98***	*E. coli *phage JS98	170,523	NC_010105	202/266	3	JS98 and JS10 share ~98% ORF homology with each other.
	*E. coli *phage JS10	171,451	NC_012741	197/265	3	

***CC31***	*E. coli *phage CC31	165,540	GU323318	156/279	8	~43% of the CC31 ORFs are unique to this phage. Also, CC31 is the only known non*T-even type *phage predicted to encode *glucosyl transferase *genes (see Table 3)

***RB43***	*E. coli *phage RB16	176,789	HM134276	115/260	2	The genomes of RB16 and RB43are similarly organized and share >85% ORF homology with each other [14]
	*E. coli *phage RB43	180,500	NC_007023	118/292	1	

***133***	*Acinetobacter *phage133	159,897	HM114315	110/257	14	Each of these *Acinetobacter *phages has a unique set of ORFs that occupy ~35% of the genome. That is, each represents a different *type *of T4-related phage genome.
***Acj9***	*Acinetobacter *phage Acj9	169,953	HM004124	97/253	16	
***Acj61***	*Acinetobacter *phage Acj61	164,093	GU911519	101/241	13	
***Ac42***	*Acinetobacter *phage Ac42	167,718	HM032710	117/257	3	

***44RR***	*Aeromonas *phage 44RR	173,591	NC_005135	118/252	17	Phages 44RR and 31 share ~98% ORF homology (and ~97% sequence identity) with each other. Also, they exhibit ~80% ORF homology with phage 25
	*Aeromonas *phage 31	172,963	NC_007022	117/247	15	

***25***	*Aeromonas *phage 25	161,475	NC_008208	116/242	13	The phage 25 genome is 11-12 kb shorter than the genome of 44RR (or 31). Also, ~14% of the phage 25 ORFs are unique to this phage.

***Aeh1***	*Aeromonas *phage Aeh1	233,234	NC_005260	106/352	23	Phages Aeh1 and PX29 share ~95% ORF homology with each other and partially overlap in host-range properties
	*Aeromonas *phage PX29	222,006	GU396103	109/342	25	

***65***	*Aeromonas *phage 65	235,289	GU459069	102/439	17	~55% of the ORFs in this genome are unique to phage 65

***KVP40***	*Vibrio *phage KVP40	244,834	NC_005083	99/381	29	Phages KVP40 and nt-1 share ~85% ORF homology with each other and partially overlap in host range properties
	*Vibrio *phage nt-1	247,144	Tulane	95/400	26	

***S-PM2***	*Marine Synechococcus *phage S-PM2	196,280	NC_006820	40/236	1	See [31] for comparisons between the marine cyano phages. Based on their diversity, each represents a different type of T4-related phage genome.
***S-RSM4***	*Marine Synechococcus *phage S-RSM4	194,454	NC_013085	41/237	12	
***Syn9***	*Marine Synechococcus *phage Syn9	177,300	NC_008296	43/226	6	
***P-SSM2***	*Marine Prochlorococcus *phage P-SSM2	252,401	NC_006883	47/329	1	
***P-SSM4***	*Marine Prochlorococcus *phage P-SSM4	178,249	NC_006884	46/198	0	

***KP15***	*Klebsiella pneumoniae *phage KP15	174,436	GU295964	116/239	1	~80% of KP15 ORFs are homologous and similarly organized to ORFs in RB43

***W14***	*Delftia acidovorance *phage phiW-14	157,486	NC_013697	60/236	0	

***AG3***	*Shigella boydii *phage phiSboM-AG3	158,006	NC_013693	64/260	4	

^(1) ^In this column, numbers with the prefixes NC, GU and HM refer to GenBank accession numbers and the designation "Tulane" refers to the database at http://phage.bioc.tulane.edu (soon to be transferred to http://phage.ggc.edu). The NC_000866 accession is for the T4 genome sequence that was compiled from data contributed by many laboratories [2,7]. The HM137666 accession is for the T4 genome sequence determined on DNA from a single source, termed T4T, which is the wild-type T4D strain maintained by the Karam laboratory at Tulane University, New Orleans.

^(2)^In this column, "% ORF homology" refers to the percentage of ORFs that are alleles between the compared genomes.

The listing shown in Table 2 should be regarded as somewhat arbitrary since setting the homology standard to a higher or lower value than ~85% can result in different groupings. In fact, as will be explained below for the *T-even type *phages, small differences in the genetic composition can have major biological consequences, which might merit further subdivisions within this cluster. In addition, as evidenced by information from the recently analyzed T4 relatives listed in Tables 1 and 2, the isolation of new T4-related phages for known and newly recognized bacterial hosts is likely to reveal a greater diversity of phage genome *types *and virion morphologies than the listing in Table 2 provides.

### Genetic commonalities between T4 relatives

A few years ago, a comparative analysis of ~15 completely or almost completely sequenced T4-related genomes showed that they share two important characteristics [14]:

1. Their genes are contained in a circularly permuted order within linear dsDNA chromosomes. In most cases, this characteristic became evident during the assembly and annotation of DNA sequence data into single contiguous sequences (contigs) and in some cases, the ends of the single contigs were further confirmed to be contiguous with each other by use of the PCR [14,17,27]

2. The genomes were each predicted to encode a set of 31-33 genes that in T4 have been implicated in the ability of the phage to exercise autonomous control over its own reproduction. This control includes the biochemical strategies that determine the circularly permuted chromosomal design, which is generated through the integration of the protein networks for DNA replication, genome packaging and viral assembly in the phage developmental program [8]. This set of genes amounts to a mere ~12% of the T4 genome.

Expansion of the sequence database to >20 different *types *of T4-related genome configurations (Table 2) has reinforced the observation that a core set of 31-33 genes is a unifying feature of all T4 relatives. However, it has also become increasingly evident that other phage genes enjoy a very wide distribution among these genomes, suggesting that the minimum number of genes required to generate a plaque-forming phage with generally similar morphology to T4 is greater than the number of the universally distributed genes and might vary with specific adaptations of different clusters of closely interrelated phages in nature. As is the case with other host-dependent, but partially autonomously replicating genetic entities in the microbial world, particularly the bacterial endosymbionts [28-30], there is usually a dependence on auxiliary functions from the entity and this dependence can vary with the host in which the entity propagates. In T4, it is already known that some phage-encoded functions are essential for phage growth in some *E. coli *strains but not others and that in many instances mutations in one gene can result in decreased dependence on the function of another gene. Many such examples of intergenic suppression have been published and referenced in comprehensive reviews about the T4 genome [2,7,8]. The analysis of the genomes of some T4 relatives has also yielded observations suggesting that ordinarily indispensable biochemical activities might be circumvented or substituted in certain genetic backgrounds of the phage or host genome. Examples include two separate instances where the need for the recombination and packaging Endonuclease VII (gp49; encoded by gene *49*), which is essential in T4, appears to have been circumvented by the evolution of putative alternative nucleases (through replacements or new acquisitions) in the *E. coli *phage RB16 (*RB16ORF270c*) and the *Aeromonas *phage 65 (*65ORF061w*) [14]. Another example is the possible substitution of the essential dUTPase function provided by gp56 in T4 by host-like dUTPase genes in the *Aeromonas *phages 65 and Aeh1 and the vibriophages KVP40 and nt-1 [14,17].

**Figure 1:**
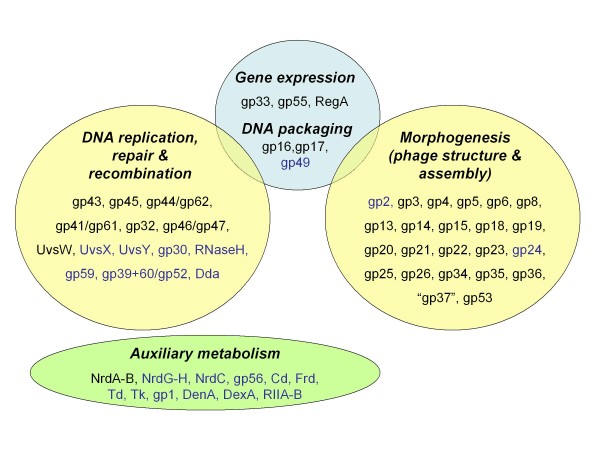
**The protein products of the Core Genome of the T4-like Viruses**. The functions of the phage gene products ("gp" designations) mentioned in this Figure are discussed in the text and summarized in **Table 3**.

Taking into consideration the distribution of T4-like genes in the >20 different *types *of phage genome configurations listed in Table 2 and the examples of putative genetic substitutions/acquisitions mentioned above, we estimate that the Core Genome of the T4-related phages consists of two genetic components, one highly resistant and one somewhat permissive to attrition in evolution. We refer to the genes that are essential under all known conditions as "Core genes" and those that can be substituted or circumvented in certain genetic backgrounds of the phage and/or bacterial host as "Quasicore genes". In Table 3 and Figure 1 we list the two sets of genes and highlight their functional interrelationships and some of the conditions under which some Quasicore genes might not be required. Interestingly, the absence of members of the Quasicore set is most often observed in the T4-related marine cyanophages, which also exhibit the smallest numbers of T4-like genes and the greatest sequence divergence in Core genes from any of the other host-specificity groups of T4 relatives listed in Tables 1 and 2. Possibly, the marine cyanobacteria represent a natural environment that has favored the evolution of a specific streamlining of the genetic background for the Core Genome of T4-related phages. This streamlining might have been driven through a combination of what the cyanobacterial hosts could provide as substitutes for physiologically important, but occasionally dispensable functions of these phages and what the phage genomes themselves might have acquired as alternatives to lost genes by LGT from other biological entities. We view each *type *of phage genomic framework listed in Table 2 as a specific adaptation of the Core Genome in the evolution of these phages in the different bacterial genera or species where T4 relatives have been detected.

**Table 3:** Genes of the Core Genome of T4-like Viruses

**T4 genes**^**(1)**^	**Gene products and/or activities**^**(1)**^	**Comments**^**(2)**^
***DNA replication, repair and recombination***		

***43*; *45*; *44 ***and ***62*; *41 *&***61; 59;****32*; *46 ***&***47*; *uvsW*; ***uvsX, uvsY; 30; rnh; 39+60 &52; dda; 49*	**gp43 (**DNA polymerase); **gp45 **(trimeric sliding clamp); **gp44/gp62 **sliding clamp loader complex (gp44 tetramer+gp62 monomer); **gp41/***gp61*helicase-primase complex (hexamers of both proteins); **gp59 **(helicase-primase loader & gp43 regulator); **gp32 **(single-strand binding protein); **gp46-gp47 **(subunits of a recombination nuclease complex required for initiation of DNA replication); **UvsW protein **(recombination DNA-RNA helicase, DNA-dependent ATPase); *uvsX *(RecA-like recombination protein); *uvsY *(uvsX helper protein); *gp30 *(DNA ligase); *Rnh *(Ribonuclease H); *gp39+60 & gp52 *(subunits of a Type II DNA topoisomerase); *Dda *protein (short-range DNA helicase); *gp49 *(Endonuclease VII, required for recombination & DNA packaging).	Many of the Quasicore genes in this group are absent in one or more T4-related marine cyanophages. In T4, some these genes are not required in certain *E. coli *hosts or become dispensable in the presence of mutations in specific other genes (intergenic suppression).

***Auxiliary metabolism***		

***nrdA ***&***nrdB***; *nrdC*; *nrdG*; *nrdH; 56*; *cd*; *frd*; *td*; *tk*; *1*; *denA*; *dexA*	**NrdA-NrdB **(subunits of an aerobic ribonucleotide reductase complex); *NrdG & NrdH *(subunits of an anaerobic ribonucleotide reductase complex); *NrdC *(thioredoxin); *gp56 *(dCTPase-dUTPase); *Cd *(dCMP deaminase); *Frd *(DHFR; (dihydrofolate reductase); *Td *(thymidylate synthetase), *Tk *(thymidine kinase); *gp1 *(dNMP kinase); *DenA *(Endonuclease II); *DexA *(Exonuclease A).	A combination of at least some of these genes is required to supplement the intracellular pool of nucleotides for phage DNA and RNA synthesis.

***Gene expression***		

***33***; ***55***; ***regA***	**gp33 **(essential protein that mediates gp55-gp45-RNA polymerase interactions in late transcription); **gp55 **(sigma factor for late transcription); **RegA **(mRNA-binding translational repressor; also involved in host nucleoid unfolding)	In T4, *regA *mutations are not lethal, yet all the T4 relatives examined so far encode homologues of this gene.

***Phage morphogenesis***		

*2*; ***3***; ***4***; ***5***; ***6***; ***8***; ***13***; ***14***; ***15***; ***16 17***; ***18***; ***19***; ***20***; ***21***; ***22***; ***23***; ***25***; ***26***; ***34***; ***35***; ***36***; "***37***"; ***49; 53***	*gp2 *(protects ends of packaged DNA against RecBCD nuclease); **gp3 **(sheath terminator); **gp4 **(Head completion protein); **gp5 **(baseplate lysozyme hub component); **gp6 **(baseplate wedge component); **gp8 **(baseplate wedge), **gp13 **(head completion protein ); **gp14 (**head completion protein); **gp15 **(tail completion protein); **gp16 **&**gp17 **(subunits of the terminase for DNA packaging); **gp18 **(tail sheath subunit), **gp19 **(tail tube subunit); **gp20 **(head portal vertex protein); **gp21 **(prohead core protein and protease); **gp22 **(prohead core protein); **gp23 **(precursor of major head protein); **gp25 **(base plate wedge subunit); **gp26 **(base plate hub subunit); **gp34 **(proximal tail fiber protein subunit); **gp35 **(tail fiber hinge protein); **gp36 **(small distal tail fiber protein subunit); **gp37 ( **large distal tail fiber protein subunit; heterogeneous among T4 relatives); gp49 (Endo VII; required for DNA packaging); **gp53 **(baseplate wedge component)	T4 gp2 is not required in recBCD mutant hosts and no gene 2 homologues are detected in some marine cyanophages. Also, the ***"37" ***designation means that in some T4 relatives (e.g. the marine cyanophages and the vibriophages), the identification of gene 37 and other tail fiber genes can be difficult or impossible to make by bioinformatic tools because of extensive mosaicism or putative substitutions with non-homologous tail-fiber genes.

*Other*		

*rIIA *&*rIIB*	The precise functions of the *rIIA *and *rIIB *gene products are not known. In T4, *rIIA *or *rIIB *mutations exhibit multiple effects on phage physiology, but are only lethal in the presence of a lambda prophage.	Like many other Quasicore genes, the *rIIA *and *rIIB *genes are found in all T4 relatives, except the marine cyanophages. The wide natural distribution of these 2 genes might be a reflection of the distribution of prophages that restrict T4 relatives in various bacterial hosts.

^(1)^Core genes and their products are shown in bold font and Quasicore genes and their products in unbolded italic.

^(2)^See text for additional explanations

### An overview of how the sequenced T4-like Viruses differ from each other

The T4-related genomes sequenced so far exhibit divergence from one another in several respects including; (a) the range of bacterial host species that the respective phages infect, (b) the sizes of these genomes and the capsids (phage heads) in which they are packaged, (c) the types of modifications, if any, that the genomic DNA undergoes in vivo, (d) their assortment of protein- and tRNA-encoding genes, (e) their assortment of T4-like genes (alleles of T4 genes), (f) the sequence divergence (mutational drift) and in some cases, the intragenic mosaicism between alleles and (e) the topological arrangement of alleles and their regulatory signals in the different genomes. Divergence between genomes within some of these categories appears to have occurred independently of other categories. For example, phages that share a bacterial host do not necessarily share similar genome sizes, similar genetic compositions at a global level, similar DNA modifications or similar genome topologies. On the other hand, phages that infect different bacterial host species seem to exhibit the highest degree of divergence from each other in most or all categories. The assignment of T4 relatives to the different groups or *types *listed in Table 2 takes into account shared similarities in most categories, the implication being that members of a phage/genome *type *are probably more closely related to each other than they are to members of other clusters of interrelated phages. For example, in pair-wise comparisons, the *T-even type *phages listed in Table 2 exhibited 85-95% genome-wide homology (shared alleles) as well as high levels of nucleotide sequence identity with each other. Most of the dissimilarities between members of this cluster of phages map to genomic segments that have long been known to be variable between T2, T4 and T6, based on electron microscopic analysis of annealed DNA mixtures from these phages [26]. Phage genome sequencing has shown that the hypervariability of these segments among all *types *of T4 relatives involves: (a) an often-observed mosaicism in tail fiber genes, (b) unequal distribution of ORFs for putative homing endonucleases, even between the closest of relatives and (c) a clustering of novel ORFs in the phage chromosomal segment corresponding to the ~40-75 kb region of the T4 genome [14-16]. The biological consequences of these genetic differences are significant [2,7,8]. Although distant relatives of the three T-even phages have been isolated that also use *E. coli *as a bacterial host (e.g. phages RB43, RB49, RB69 and others; Table 2), no close relatives of these canonical members of the T4-like Viruses genus have yet been found among the phages that infect bacterial hosts other than *E. coli*. By using the ORF composition of the T4 genome as a criterion, we estimate that the range of homology to this genome (i.e., percentage of T4-like genes) among the coliphage relatives analyzed so far is between ~40% (for phage RB43) and ~78% (for phage RB69). Among the T4 relatives that grow in bacterial hosts other than the Enterobacteria, the homology to the T4 genome ranges between ~15% T4-like genes in the genomes of some marine cyanophages and ~40% T4-like genes in the genomes of some *Aeromonas *and *Acinetobacter *phages (Table 2). These homology values reflect the extent of the heterogeneity that exists in the genetic backgrounds of the two components of the Core Genome (Figure 1, Table 3) among the different phages or phage clusters listed in Table 2. The five *types *of genome configurations currently catalogued among the T4-related marine cyanophages (Table 2) range in size between ~177 kb (for phage Syn9) and ~252 kb (for phage P-SSM2) and carry the smallest number of T4-like genes among all currently recognized *types *of T4 relatives. The range here is between 40 (for S-PM2) and 47 (for P-SSM2) T4-like genes per genome [31]. A comprehensive listing of T4 alleles in most of the phages listed in Tables 1 and 2 can be found in Additional file 1 or online at http://phage.bioc.tulane.edu and http://phage.ggc.edu. The recent genome entries in GenBank mentioned earlier for phiSboM-AG3 and phiW-14 predict ~60 T4-like genes, mostly Core and Quasicore genes, for each. Taken together, these observations are consistent with the notion that components of the Core Genome have been somewhat resistant to dispersal in evolution, but that the host environment must also play an important role by determining the most appropriate genetic background of this unifying feature of T4-related genomes.

### Genome size heterogeneity among T4 relatives

**Figure 2:**
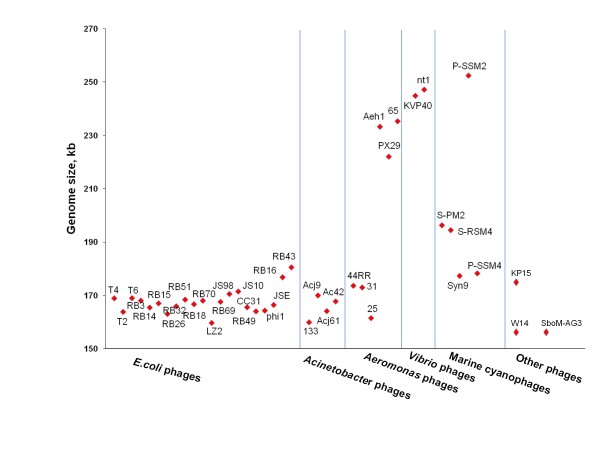
**Distribution of genome sizes among the sequenced T4 related phages (Table 2)**. The graphic highlights the distribution of phage genome sizes (red diamond shapes) in each of the bacterial host-specificity domains from which T4-related phages have been isolated (**Table 1**).

In Figure 2 we show a graphic representation of the heterogeneity in genome sizes for the phages listed in Table 2. The size range observed so far for genomes of the T4-like Viruses is between ~160,000 and ~250,000 bp (or ~160-250 kb). Relatives of T4 with genomes near or larger than 200 kb also exhibit larger and more elongated heads than phages with genomes in the ~170 kb size range [9,10]. These extraordinarily large T4 relatives have sometimes been referred to as "Schizo T-even" phages [32] and rank among the largest known viruses, i.e., the so-called "giant" or "jumbo" viruses [33]. T4-related giants have been isolated for *Aeromonas, Vibrio *and marine cyanobacterial host species, but no such giants have yet been isolated for T4 relatives that grow in *E. coli *or the other host species listed in Table 1. For the *Vibrio *bacterial hosts, only giant T4 relatives have been isolated so far, whereas a wide range of phage genome sizes has been observed among the *Aeromonas *and cyanobacterial phages. Comparative genomics has not yet revealed any genetic commonalities between the T4-related giant phages of *Aeromonas, Vibrio *and marine bacteria (Fgure 1) that might explain the cross-species similarities in head morphology. So, it remains unclear what might have determined the evolution of different stable genome sizes in different phage lineages or clusters. It is equally possible that giant genomes can evolve from smaller precursors or can themselves serve as progenitors of smaller genomes. Detailed studies of the comparative genomics of the functional linkage between DNA replication, packaging and morphogenesis for the different genome size categories shown in Figure 2 might be needed to provide explanations for what determines the evolution of different genome sizes in different phage clusters or lineages. Also, fine-structure morphological differences do exist among T4 relatives that are of similar size and share homologies for structural genes, indicating that the determination of head size and shape can vary with different combinations of these genes.

Some observations in the T4 biological system further underscore the plasticity of head-size determination and the dependence of this plasticity on multiple genetic factors in phage development [8]. Based on mutational analyses, the interplay of at least four T4 genes can generate larger (more elongated) phage heads containing DNA chromosomes that are larger than the ~169 kb size of wild-type T4 DNA. These are the genes for the major capsid protein (gene *23*), portal protein (gene *20*), scaffold protein (gene *22*) and vertex protein (gene *24*). In addition, the recombination endonuclease Endo VII (gp49) and the terminase (gp16 and gp17) play important roles in determining the size of the packaged DNA in coordination with head morphogenesis (headful packaging). Possibly, it is the regulation of these conserved gene functions that can diverge coordinately with increased genetic acquisitions that lead to larger genomes and larger heads in certain cellular environments. The T4-related *Aeromonas *phages would be particularly attractive as experimental systems to explore the evolutionary basis for head-genome size determination because this subgroup of phages is easy to grow and contains representatives of the entire range of phage genome and head sizes observed so far (Figure 2 and Table 2).

### Lateral mobility and the Core Genome of the T4-like Viruses

It is clear that the Core Genome of the T4-related phages has spread to the biological domains of a diversity of bacterial genera (Table 1), although it is unclear how this spread might have occurred and to what degree genetic exchange is still possible between T4 relatives that are separated by bacterial species barriers and high sequence divergence between alleles of the Core and Quasicore genes listed in Table 3 and Figure 1. Such exchange would require the availability of mechanisms for transferring Core Genome components from one bacterial species domain into another. In addition, shuffled genes would have to be compatible with new partners. Experimentally, there is some evidence indicating that the products of some Core genes, e.g., the DNA polymerase (gp43) and its accessory proteins (gp45 and gp44/62), can substitute for their diverged homologues *in vivo *[12,34-36]. Such observations suggest that the shuffling of Core Genome components between diverged T4 relatives can in some cases yield viable combinations. However, for the most part there appear to be major barriers to the shuffling of Core Genome components between distantly related T4-likeViruses in nature. In some respects, the mutational drift within this common core should provide valuable insights into its evolutionary history since the last common ancestor of the T4 related genomes examined so far [19,20]. On the other hand, it should be recognized that the evolutionary history of the Core Genome is not necessarily a good predictor of whole phage genome phylogeny because the majority of the genetic background of this common core varies considerably between the different *types *of T4 relatives (Table 2) and is probably derived from different multiple sources for different phage lineages or clusters.

Although the Core Genome of the T4-related phages might resist fragmentation in evolution, it is unclear if there could have been one or more than one universal common phage ancestor for all of the genes of this unifying feature of the analyzed T4 relatives. Some answers about the origins of the different multi-gene clusters that constitute the Core Genome of these phages might come from further exploration of diverse environmental niches for additional plaque-forming phages and other types of genetic entities that might bear homologies to the Core and Quasicore genes (Table 3 and Figure 1). For example, it remains to be seen if there are autonomously replicating phages or plasmids in nature that utilize homologues of the T4 DNA replication genes, but lack homologues of the DNA packaging and morphogenetic genes of this phage. Conversely, are there phages in nature with alleles of the genes that determine the T4 morphotype, but no alleles of the T4 DNA replication genes? The natural existence of such biological entities could be revealed through the use of the currently available sequence database for T4-related genomes to design appropriate probes for metagenomic searches of a broader range of ecological niches than has been examined so far. Such searches could be directed at specific Core or Quasicore genes [37] or specific features of the different *types *of phage genomes listed in Table 2. It is worth noting that putative homologues of a few T4 genes have already been detected in other genera of the Myoviridae, e.g. the *Salmonella *phage Felix 01 (NC_005282) and the archaeal *Rhodothermus *phage RM378 (NC_004735). Both of these phages bear putative homologues of the T4 gene for the major capsid protein gp23. So, it appears that at least some of the Core and Quasicore genes of the T4-related phages (Figure 1, Table 3) can survive lateral transfer and function in genetic backgrounds that lack homologies to their presumed ancestral partner genes. In addition, a very recent report [38] describes two *Campylobacter *phages (CPt10 and CP220) that appear to be related to T4, based on the large number of putative T4-like genes that they bear (see GenBank Accession nos. FN667788 and FN667789). Other recent submissions to GenBank that deserve attention and further analysis include the genomes of *Salmonella *phage Vi01 (FQ312032), and *E. coli *phage IME08 (NC_014260; an apparent close relative of phage JS98). Clearly, the sequence database for T4-related genomes requires further enhancements and detailed EM characterization of all of the sequenced phages is needed before a clear picture can emerge about the contributions of the host or host ecology to evolution of the genetic framework and morphological fine-structure within the extended family of T4 relatives.

**Figure 3:**
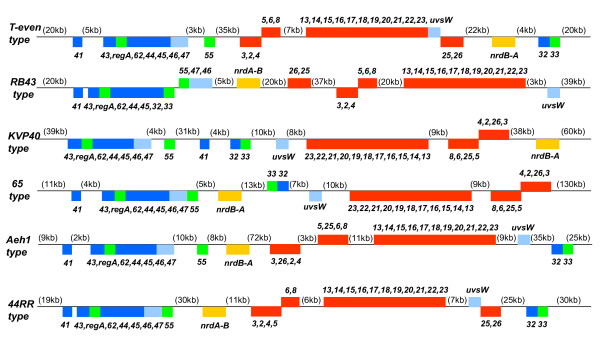
**Divergence of the organization of Core genes among different *types *of T4-related genomes**. The numbers and acronyms shown alongside the color-coded bars refer to the names of the phage-encoded genes and proteins listed in Table 3, which also summarizes their specific biochemical roles. DNA replication genes are color-coded dark blue, the recombination/repair genes light blue, the transcription and translation genes green, the morphogenetic genes red and the genes for aerobic nucleotide reductase (*nrdAB*) orange.

Additional evidence suggesting that some Core Genome components of T4 relatives can be subjected to lateral transfer in natural settings comes from the variety of topologies (different genetic arrangements) that have been observed for the Core genes in the phages analyzed so far. In Figure 3, we show six examples of naturally existing topologies for the set of Core genes listed in Table 3. The topology exhibited by the *T-even type *phages is shared by the majority of the other T4-related *E. coli *phages and by all 4 of the T4-related *Acinetobacte*r phages listed in Table 2. Interestingly, the two *E. coli *phages RB16 and RB43 exhibit a unique genome topology that has most of the DNA replication genes clustered together in one genomic sector. This *RB43 type *topology is also observed in the recently annotated genome of *Klebsiella *phage KP15 (as we surmise from by our own examination of GenBank Accession no. GU295964). Interestingly, the RB16 and RB43 genomes are rich in a class of putative homing endonuclease genes (HEGs) that bear sequence similarities to the genes for a class of DNA-binding proteins that mediate genetic rearrangements in the developmental programs of plants [14,39-41]. The other unique genome topologies shown in Figure 3 have been observed for the *Vibrio *phage KVP40 (and its close relative nt-1) and several *Aeromonas *phages, including the giant phages 65 and Aeh1 (and its close relative phage PX29) and the smaller phages 25 and 44RR (and its close relative phage 31), respectively. The marine cyanophages exhibit yet other topologies for Core Genome components [31,42]. The diversity of Core Genome topologies underscores the ability of Core and Quasicore genes to function in different orientations and in a variety of genetic backgrounds and regulatory frameworks [14]. The genetic regulatory sequences for a number of Core genes, like phage replication genes *43 *(DNA polymerase) and *32 *(Ssb protein), are highly diverged between representatives of the different *types *of T4 relatives listed in Table 2[14], further reflecting the adaptive potential of the T4-related Core Genome. Another indication that this genetic core can be prone to lateral transfer is the observed colonization of some of the Core or Quasicore genes or their vicinities by mobile DNA elements, especially intron-encoded and freestanding HEGs [14,43,44]. We will discuss the possible roles of these elements in the evolution of T4-related genomes later in this review.

### The Pangenome of the T4-like Viruses

Collectively, the genetic backgrounds for the Core Genome of the T4 relatives examined for the current report are predicted to encode a total of ~3000 proteins that do not exhibit statistically significant sequence matches to any other proteins outside of the databases for the T4-related phages. This number of ORFs is ~1.5 orders of magnitude larger than our estimate of the number of Core plus Quasicore genes in the Core Genome of these phages (Figure 1, Table 3), and might be several orders of magnitude smaller than the union of all the different ORFs that exist in T4-related phages in nature. We refer to this union as the "Pangenome" of the T4-like Viruses, in analogy to the pan genomes of other known groups of autonomously replicating organisms [30]. Based on results from the recent isolation and analysis of the T4-related coliphage CC31 and the *Acinetobacter *phages Acj9 and Acj61 listed in Table 2 , novel and highly divergent members of the T4-like Viruses might be easily detected in environmental samples by taking advantage of the bacterial host diversity of these phages, the uniqueness of certain sequences in specific phage genomes or lineages and other characteristics that distinguish between the different clusters or *types *of phage genomes listed in Table 2. The analysis of the genomes of phages CC31, Acj9 and Acj61, predicted that each encodes ~120 newly recognized ORFs that can be added to the growing count of the Pangenome of the T4-like Viruses (unpublished observations). Such observations suggest that additional diversity is likely to be uncovered through the isolation and analysis of larger numbers of T4 relatives for the known as well as previously unexplored potential bacterial hosts of these phages [38,45].

Despite their plasticity in genome size and their increasing inventory of new ORFs, there are indications that natural diversity of the T4-related phages is not unlimited. We already know of pairs and triplets of nearly identical (yet distinct) genomes that have been isolated years apart from each other and from different geographical areas (Tables 1 and 2). The natural existence of such nearly identical phage genomes might mean that there are limits to the number of genetic backgrounds that can evolve around a certain Core Genome composition. The limitations might be imposed by the specific partnership that an evolving phage ultimately establishes with its bacterial host(s). More examples of nearly identical genomes in nature would be desirable to find since they might provide clues to the incremental changes by which progenitor genomes can begin to branch into different lineages through additions, deletions and exchanges in the genetic background of the Core Genome.

### Genetic isolation between T4 relatives

Genetic separation between interrelated phages can evolve within a shared bacterial host range, as for example might have occurred for the *E. coli *phages T4 and RB69 [25] or come about as a consequence of the transfer of the capacity for whole genome propagation from one host species to another, as might be represented by the different host-specificities of the phages listed in Tables 1 and 2. Insights into the biochemical processes that might lead to the genetic isolation of a T4-related genome from close relatives can be drawn from the number of studies that have been carried out on phage-phage exclusion and host-mediated restriction of the T-even phages [8,46,47]. As explained below, the three T-even phages and their close relatives (*T-even type *phages, Table 2) represent a scenario in which small changes in a genome might result in major effects on its compatibility with a parental genotype.

Phages T2, T4 and T6 can undergo genetic recombination and phenotypic mixing with each other *in vivo *(in pair-wise co-infections of their shared *E. coli *hosts), but they are also partially incompatible with each other under these conditions [11]. The genomes of these phages encode similar, but distinct enzyme networks that modify their genomes and prevent their restriction by gene products encoded by the bacterial hosts and/or certain prophages or defective prophages that can reside in some of these hosts [46,47]. In addition, a few genetic differences between these otherwise closely interrelated phages cause them to be partially incompatible. The genes known to be involved in T-even phage genome modification and restriction are listed in Table 4. Some of these genes specify the modification of phage genomic DNA with glucosylated hydroxymethyl (gluc-Hm) groups at dCMP residues, whereby the DNA becomes resistant to host restriction activities, particularly the *E. coli *Mcr (Rgl) enzyme system. Other phage genes are responsible for commandeering the host transcription system for expression of the modified phage DNA and away from the expression of any DNA (including the host genome) that does not carry the phage-induced modifications [8,48,49]. Subtle differences in phage DNA modification and the interplay between phage- and host-encoded proteins can limit the opportunities for genetic recombination between the very similar phage genomes.

**Table 4:** Distribution of alleles of the T4 DNA modification, restriction and antirestriction genes in T4-related phages^(1^^)^

T4 Gene	Product	Role	Phages with alleles of the T4 gene
*42*	dCMP - hydroxymethylase	Hm-dCMP synthesis	All *T-even type *and *JS98 type *phages. Also phages RB69 and CC31, all 4 *Acinetobacter *phages (133, Acj9, Acj61 and Ac42) and the *Aeromonas *phages 44RR, 31 and 25.

*56*	dCTPase - dUTPase	Increases dCMP pool, decreases dCTP pool; provides dUMP for dTMP synthesis	All phages listed in Table 2, except the giant *Aeromonas *phages Aeh1 and 65 and the giant *Vibrio *phages KVP40 and nt-1.

*α-gt*	α glucosyl transferase	α glucosylation of Hm-dCMP DNA	*T-even type *phages only

*β-gt*	β glucosyl transferase	β-glucosylation of Hm-dCMP DNA	Phages T4 and CC31 only

*βα-gt*	β-1, 6-glucosyl-α- glucose transferase	β glucosylation of α-glucosylated Hm-dCMP DNA	All T-even type phages, except T4; also present in CC31

*denA*	Endonuclease II (Endo II)	Limited cleavage of unmodified (dCMP-containing) DNA	All T4 relatives, except the *Acinetobacter *phages and marine cyanophages listed in Table 2

*denB*	Endonuclease IV (Endo IV)	Extensive cleavage of unmodified (dCMP containing) DNA	Same distribution as gene *42*

*alc*	Alc protein	Disallows transcription of unmodified (dCMP containing) DNA	Same distribution as gene *42*

*arn*	Arn protein	Counters the restriction effects of the host McR (Rgl) system	All *T-even type *phages; and phage CC31 only.

(1) The information in this Table is for the phages listed in Table 2.

T2, T4 and T6 encode homologous dCTPase-dUTPase (gp56; gene 56), dCMP-hydroxymethylase (gp42; gene 42) and dNMP kinase (gp1; gene 1) enzymes that together create a pool of hydroxymethylated-dCTP (Hm-dCTP) for phage DNA synthesis. The Hm-dCMP of the synthesized DNA is further modified by the addition of glucose molecules to the Hm groups. The glucosylation is carried out differently and to different extents between the three phage relatives. They all encode homologues of an α-glucosyltransferase (*αgt *gene) that adds glucose molecules to the Hm groups in the α-configuration; however, the T2 and T4 enzymes glucosylate 70% whereas the T6 enzyme glucosylates only 3% of these groups in the respective genomes. The three phages also differ in a second wave of glucosylations of the genomic Hm-dCMP. T4 encodes a β. glucosyltransferase (*βgt *gene) that adds glucose (in the β-configuration) to the rest of the unglucosylated Hm-dCMP residues in the phage DNA, whereas T2 and T6 lack a βgt gene and instead encode a β-1,6-glucosyl-α-glucose transeferase (*βαgt *gene) that adds glucose to the glucose moieties of some of the preexisting α-glucosylated Hm-dCMP residues, thus resulting in modification of the respective Hm-dCMP residues with gentobiose. This second glucosylation occurs at 70% of the α-glucosylated residues in T2 as compared to only ~3% of these residues in T6. That is, ~25% of the Hm-dCMP residues in T2 and T6 remain unglucosylated. Enzymes of the bacterial host synthesize the UDP-glucose (UDPG) used for the glucosylation reactions by the phage-induced enzymes. Interestingly, all of the close relatives of the T-even phages listed in Table 2 (*T-even type *phages) are predicted to encode *αgt *and *βαgt *genes, *i.e., *they are similar to T2 and T6 in their glucosylation genes. However, the glucosylation patterns of these relatives have not been analyzed. Also, it is worth noting that currently, T4 is the only member of the T4-like Viruses genus known to encode α- and β-glucosyltransferases. A distant relative of the *T-even type *phages, the coliphage CC31 (GU323318), is predicted to encode the unique combination of *βgt *and *βαgt *genes and currently, is the only other phage besides T4 in which a *βgt *gene has been detected by bioinformatic analyses.

Differences in DNA modification patterns, such as those that exist between the three T-even phages might open windows for phage-encoded nucleases that are able to distinguish between their own genomes and the genomes of dissimilarly modified close relatives. Also, as has been observed in T4, a lack of Hm-dCMP glucosylation can render the Hm-dCMP-containing phage DNA susceptible to the host-encoded Mcr (Rgl) restriction system, as well as the restriction systems of some prophages that can reside in *E. coli *or other potential Enterobacterial hosts [46,47]. Possibly, the unglucosylated Hm-dCMP sites in the T2 and T6 genomes escape restriction activities originating from the host through protection by the DNA modifications in their vicinity or through evolutionary adjustments in the expression of phage genes that control the susceptibility of phage DNA to the host-encoded restriction activities. In T4, the gene *2 *protein (gp2), which attaches to DNA ends, protects against degradation by the host RecBCD exonuclease (Exo V) and the *arn *gene product (Arn protein) protects unglucosylated Hm-dCMP DNA against the host Mcr system [50-52] (Table 4). It would be interesting to find out if the *arn *gene and gene *2 *are controlled differently in the different *T-even type *phages. All the phages in this cluster are predicted to encode homologues of T4 genes *56, 42, 2 *and *arn *(Table 4) and at least some of them exhibit partial mutual exclusion with the T-even phages [25]. Elucidating the molecular basis for the partial incompatibilities within this cluster of closely interrelated phages might shed light on some subtle differences in phage genome adaptation that can begin to transition close relatives towards total genetic isolation from each other.

Additional factors that can potentially contribute to phage-phage exclusion between relatives that share the same bacterial host are the products of phage-specific nuclease genes, some of which might be imported into evolving phage genomes through lateral DNA transfer. Among these are genes for homing enzymes (HEGs), which exist as different types and in variable numbers among T4-related phage genomes. At least three HEG-encoded nucleases have been implicated in the partial exclusion of T2 by T4 [53-55]. Other types of inhibition of one T4-related phage by another are also possible and might potentially be discovered among the predicted products of the numerous novel ORFs in the Pangenome of the T4-like Viruses. The distribution of HEGs in the genomes of the phages listed in Tables 1 and 2 is discussed later in this review.

There are some distant relatives of the T-even phages that encode homologues of genes *42 *and *56*, but that lack homologues of the glucosyltransferase genes. Examples are the coliphages RB69 and JS98 and the *Aeromonas salmonicida *phages 44RR, 31 and 25 (see Table 2 for GenBank Accession nos.). These gene *42*-encoding phages also encode homologues of the T4 genes that have been implicated in phage-induced degradation or inhibition of the expression of unmodified (dCMP-containing) DNA, i.e., the *alc, denA *and *denB *genes (Table 4). It is not yet known if phages like RB69 and JS98 are adapted to having Hm-dCMP instead of glucosylated Hm-dCMP in their DNA (e.g., through effective inhibition of the host restriction systems) or if they encode other types of modifications to the Hm-dCMP residues that provide similar protection from restriction by the host as does the glucosylation in *T-even type *phages. In addition, there are many T4 relatives that lack homologies to the entire gene network that controls DNA modification and expression of glucosylated DNA in phage T4, including genes *42 *and *56*, the glucosyl-transferase genes and the *arn, alc *and *denB *genes. The dCMP of the genomes of these phages probably lacks major modifications, as suggested by studies that have demonstrated a sensitivity of some of these genomes to certain Type II restriction endonucleases that fail to digest wild-type (modified) T4 genomic DNA [56]. Elucidation of the host-phage interactions that allow these seemingly unmodified phage genomes to propagate without being restricted by their hosts would be important for developing a better understanding of how the Core Genome of the T4-related phages has succeeded in spreading across bacterial species barriers in nature.

One example of a total incompatibility between phage T4 and a relative that also grows in *E. coli *is the exclusion of T4 by phage RB69 [25]. The T4 and RB69 genomes are >75% homologous over very long stretches of their genomes, but when introduced into the same host cells they generate no viable phage recombinants between them and only RB69 phage progeny are made. The sequencing of the RB69 genome has revealed considerable divergence in the nucleotide sequences of most of its alleles of T4 genes. So, it is not surprising that the T4 and RB69 have not been observed to exchange DNA through homologous recombination [12,35]. However, the sequence divergence between the two genomes does not explain why RB69 completely excludes T4 [25]. Interestingly, the RB69 genome is predicted to lack HEGs whereas T4 is predicted to encode many such nuclease genes. Yet, it is T4 rather than RB69 that suffers exclusion by its relative. The six *types *of T4-related phages that can grow in *E. coli *(Table 2) could potentially serve as excellent sources of material for studies of the multiple factors that can transition T4-related genomes from partial to total genetic isolation from each other despite access to the same bacterial host domain. Technological developments in DNA and genome analysis since the early studies on T4-related phage-phage exclusion should make it possible to develop PCR-based high-throughput methodologies for examining large populations of phage progeny from crosses between compatible, partially compatible or incompatible phages.

### Agents of lateral DNA transfer in T4-related genomes

Although horizontal DNA transfer is suspected to play a major role in the evolution of the T4-related phages, particularly in diversification of the Pangenome of these phages, there are few clues about the agents that might mediate such transfer. Typically, the junctions between Core Genome components and adjacent DNA presumed to be imported by lateral transfer show no similarities to the familiar sequence signatures of known bacterial mobile elements that insert through site-specific and transpositional recombination [57]. Ectopic insertions (DNA additions) and illegitimate reciprocal or nonreciprocal recombination (DNA replacements) in the natural pools of evolving T4-related phages are possible causes for diversification of phage genomes through DNA rearrangements [58,59]; however, it is unclear if such events are more likely to occur in dsDNA phage evolution (or the evolution of the T4-like Viruses in particular) than in the evolution of bacterial and other cellular genomes in the microbial world. The diversity observed among the T4-related genomes examined so far appears to be of a similar magnitude to the diversity seen between distantly interrelated bacterial genera [60]. For example, in Aeh1, KVP40 and the cyanobacterial phages (Table 2), >85% of the genetic composition is unique to the *type *of T4-related phage genome and presumed to have originated through DNA rearrangements that assembled these genomes from core and variable components. The plasticity of genome size and the ability of modules of Core genes to function in a variety of orientations and genetic neighborhoods (Figure 3) suggest that genomes of the T4-like Viruses are particularly receptive to genetic gains and losses that might improve their adaptation to new environments. In addition, based on studies with T4 [8,61], these genomes are predicted to encode a highly active enzyme system for homologous recombination that has evolved to be an integral part of the machinery for genome replication, maintenance and packaging. It is known that the enzymes for homologous recombination can also mediate non-homologous (or "illegitimate") exchanges between marginally similar or even dissimilar genetic sequences in all DNA-based biological systems. An evolving T4-related genome might incorporate foreign DNA through at least two pathways that involve illegitimate recombination; (a) traditional reciprocal exchanges with foreign genetic entities (genetic replacements) and (b) initiation of DNA replication through the invasion of intracellular phage DNA pools by free 3' ends of foreign DNA (genetic additions; see also [8]). The production of viable phage recombinants by way of such events might be rare, but the observed mosaicism between the known T4-related phages is clear evidence that genetic shuffling has been rampant in the evolution of these phages.

### Homing endonucleases as possible mediators of T4-related genome diversification

Other agents that might facilitate the acquisition of novel DNA into evolving T4-related genomes are the DNA endonucleases, especially homing endonucleases. Homing enzymes have been experimentally shown to mediate the unidirectional transfer of DNA between closely related T4-like genomes in two types of scenarios, intron homing [43,44] and intronless homing [53,54]. Both types of homing utilize homologous recombination between phages co-infecting the same bacterial host to complete the transfer of genetic information from the endonuclease-encoding genome to a recipient genome that lacks the gene for the endonuclease. In Table 5, we summarize the distribution of putative HEGs among the T4-related genomes sequenced so far. The abundance and variable distributions of these genes in this pool of interrelated phage genomes suggests that T4 and its relatives are attractive natural homes for this category of transposable elements. Also, as indicated in Table 5, most of the known or predicted HEGs in these phages exist as freestanding ORFs in the phage genomes. There are only three HEGs known that reside inside self-splicing group I introns and that have been experimentally implicated in intron homing [62]. All three reside in the cluster of *T-even type *phages [63] and have probably spread within this cluster in natural settings. In contrast, there is no convincing evidence that these elements have moved across the bacterial species and genera that separate the different clusters or phage/genome *types *listed in Table 2. Nevertheless, recently observed novel activities of HEGs suggest that this category of transposable genes might be capable of generalized transposition without leaving traces of their involvement in the lateral transfer.

**Table 5:** Distribution of HEGs or putative HEGs in sequenced T4-related genomes

Category and number of HEGs found									
**Phage genome analyzed**	***seg-likeGIY-YIG***	**other*GIY-YIG***	***mob*-like*HNH***	***HNH-AP2***	**other *HNH***	***hef*-like**	**Intron encoded*GIY-YIG HNH***		**Total**

**T4**	7		5				2	relic	15
**T2**					1				1
**T6**	6		3		1		1		11
**RB3**	4		2				1	1	8
**RB14**	2								2
**RB15**	1		1						2
**RB18**	2								2
**RB26**									None
**RB32**	2		2						4
**RB51 or RB70**	2		1		1				4
**LZ2**	3						1		4

**RB69**									None

**RB49**				1	1				2
**phi-1**	1				1				2
**JSE**	1		2	2	2			relic?	7

**JS98**									None
**JS10**									None

**CC31**									None

**RB43**			1	3					4
**RB16**	2	1	1	6					10

**133**	2	1	1			1			5
**Acj9**	2		1		1	1			5
**Acj61**	3								3
**Acc42**	4	1							5

**44RR**									None
**31**									None

**25**	2		1		1				4

**Aeh1**			1						1
**PX29**	2								2

**65**			1						1

**KVP40**		1							1
**nt-1**	1								1

**S-PM2**									None
**S-RSM4**									None
**Syn9**		1							1
**P-SSM2**		1							1
**P-SSM4**									None

**KP15**	1			1					

**W-14**					1				1

**Sbo-AG3**			2						2

In both intron-homing and intronless-homing the primary role of the homing endonuclease is to introduce a dsDNA break in the genome destined to receive the HEG-containing intron or freestanding HEG. It is the repair process for the dsDNA break that ultimately provides a copy of the donor DNA for recombination into the recipient through a gene conversion event. In this regard, any endonuclease that creates dsDNA breaks might be a potential mediator of lateral DNA transfer [64,65]. Since the enzymes for homologous recombination can mediate exchanges between marginally similar or even dissimilar sequences, it is possible that a variety of endonucleases can initiate illegitimate genetic exchanges.

There are at least three examples of freestanding HEGs in T4-related phages that are suspected to encode the homing enzymes for introns lacking HEGs of their own [36,55,65]. The natural existence of such HEGs raises the possibility that some homing enzymes can mediate the transposition of DNA that is distantly located from their own structural genes without necessarily co-transferring the HEG itself. Such a role for HEGs would be consistent with the observation that much of the mosaicism between T4-related genomes is usually not associated with closely linked HEGs; however, no experimental evidence is currently available in support of the notion that HEGs can create mosaicism at distant genetic loci. Considering the wide distribution of HEGs in what is probably only a small sampling of the diversity of T4-related genomes in nature, this class of genomes might ultimately prove to be a rich repository of other as yet unidentified families of HEGs.

It is perhaps not surprising that introns appear to be much less abundant than HEGs in T4-related genomes. To persist in evolution, introns must be able to guarantee the survival of their host by maintaining their self-splicing activities. Introns depend on homing enzymes for their spread, although they can integrate less frequently through reverse splicing [66,67]. In contrast, untranslated intercistronic regions offer a much larger selection of potential targets for the insertion of HEGs, which might also enter genomes through rare ectopic insertion [68]. The three group I introns that have been described for the *T-even type *phages all encode their own HEGs , i.e., the introns in the *td *(I-TevI), *nrdB *(I-TevII) and *nrdB *(I-TevII) genes (Table 5). A fourth group I intron was recently described for the DNA polymerase gene (gene *43*) of the *Aeromonas salmonicida *phage 25 (Intron *25.g43B*) [36]. This intron lacks its own HEG, but is predicted to use a freestanding HEG for mobility. Another putative group I intron can be detected in gene *43 *of the recently published genome sequence of phage JSE, a close relative of phage RB49 [69]. Our own examination of this sequence suggests that the JSE intron contains a truncated derivative of a former HEG, i.e., much like the existence of a truncated HEG in the intron of the T4 *nrdB *gene [70]. Such HEG truncations might add to the difficulties in detecting traces of these mobile elements in contemporary phage genomes.

In summary, the observations cited above suggest that the self-mobilizing freestanding HEGs are potential agents of lateral transfer that might contribute to genomic mosaicism by mobilizing a variety of genetic sequences in phage genomes, including introns and flanking as well as distant DNA and genes or gene clusters.

### Concluding remarks

Genomes of the T4-like Viruses are repositories of a diversity of genes for which no biological roles have been assigned or can be predicted on the basis of comparisons to other sequences in databases. The reference for these phages, phage T4, has been extensively studied [2,7,8] and provides a rational basis for suspecting that the diversity among its relatives is a reflection of adaptations of a core phage genome to a variety of challenges in evolution, including encounters with new host environments. Experimentally, many T4 genes that are not essential for phage propagation in some bacterial hosts or genetic backgrounds are nevertheless essential in others (see [8] for examples). Bacterial genomes are themselves dynamic entities that are subject to the trafficking of prophages, plasmids and possibly other entities that can restrict or complement the propagation of other invaders of bacteria. There are at least three examples in the T4 biological system where prophages or defective prophages can restrict T4 phage growth. These are the restriction of T4 *rII *mutants by lambda lysogens, the restriction of unglucosylated HMC-DNA by P1 lysogens and the restriction of late phage gene expression by the e14 element [8]. Such examples underscore the important role that the host (and its resident prophages) must play in determining the T4-related genotype required for survival in the host environment. The range of natural bacterial hosts for any of the phages listed in Tables 1 and 2 might be much broader than what is available or has been used in laboratories to propagate these phages and evaluate their physiology. The isolation of new T4 relatives for known bacterial hosts as well as the identification of new bacterial hosts for known and new types of T4-related phages would be important for bridging the many gaps in our understanding of how the T4-like Viruses have managed to spread across bacterial species barriers. At the very least, the current sequence database for these Myoviridae should prove to be a rich source of genetic markers for bioprospecting as well as being a mine of reagents for basic research and biotechnology.

In regard to studies of the basic mechanisms of molecular evolution, the T4-like Viruses constitute a large pool of interrelated autonomously replicating entities that are highly accessible to analysis of broadly applicable concepts in biology. The genomes of these viruses are large by viral standards and exhibit many parallels to the mosaicism and diversity of prokaryotic cellular genomes. The phage genomes analyzed so far (Table 2) could be used as reference points for the analysis, especially through metagenomic tools, of large populations of closely interrelated phages within specific ecological domains without having to isolate these phages as plaque-forming units. This would be particularly important for the detection of commonalities between T4-related genomes and other types of genomes in the microbial world. In addition, such metagenomic approaches would be useful for detecting the continuities and abrupt discontinuities that occur at the branch points between phage lineages.

As potential sources of interesting gene products for studies of biological structure and function, one needs only to scan the literature for the numerous examples where T4-encoded proteins have been used to elucidate the mechanisms of processes common to most organisms, such as DNA replication, transcription, translation, genetic recombination, mutation, homing and others. One of the most important paths to biological diversification is the path to changes in the specificities of proteins and nucleic acids that retain their essential biochemical activities. The collection of sequenced T4-related phages is already a rich source of such examples of diversification of protein specificity.

Finally, we should mention the resurgence of interest in bacterial viruses as sources of toxins [71] and as potential therapeutic agents against bacterial pathogens [72,73]. T4 and its known relatives are classical examples of how virulent a virus can be against one bacterial host and ineffective against many other bacteria. These phages have no other lifestyle but the one leading to cell death and they use multiple targets in their attacks on hosts. The different specificities with which the T4-like Viruses recognize and inhibit different bacterial host species raise hopes that phage-induced gene products can be found that are highly specific to targets in specific bacterial pathogens. By using combinations of these gene products to attack multiple targets the development of bacterial resistance against these biological drugs would become highly unlikely. Bacteriophage genomics and particularly the genomics of T4-related phages are opening windows to many new frontiers of basic and applied biology.

## List of Abbreviations

contigs: Contiguous sequences; dsDNA: Double-stranded DNA; HEG: Homing endonuclease gene; Hm: Hydroxymethyl; ICTV: International Committee for the Taxonomy of Viruses; LGT: Lateral gene transfer; ORF: Open-reading frame; PCR: Polymerase chain reactions; UDPG: Uridine diphosphate-glucose

## Competing interests

The authors declare that they have no competing interests.

## Authors' contributions

J. D. Karam wrote the first draft of the manuscript with considerable help from V. Petrov and S. Ratnayaka, who prepared summaries for the Tables and Figures. Also, V. Petrov had participated heavily in the analysis of a large number of the genomes reviewed here and prepared most of the genomes sequenced in the Karam laboratory for submission to GenBank. S. Ratnayaka assisted V. Petrov in these efforts. J. Nolan created and manages the websites http://phage.bioc.tulane.edu (more recently http://phage.ggc.edu), which was used extensively in the preparation of the summaries presented in this review. J. Nolan also contributed unpublished information about the sequences of several close relatives of T4 and he and E. Miller contributed numerous suggestions for improvement of the manuscript. In addition E. Miller facilitated the sequence analysis of a number of the phage genomes discussed here. All authors read and approved the final manuscript.

## Supplementary Material

Additional file 1 **Table S1**. A comprehensive listing of T4 alleles in most of the phages listed in Tables 1 and 2 can be found in Additional file 1 Click here for file
